# Ultrathin 2D Cobalt Zeolite‐Imidazole Framework Nanosheets for Electrocatalytic Oxygen Evolution

**DOI:** 10.1002/advs.201801029

**Published:** 2018-10-13

**Authors:** Kolleboyina Jayaramulu, Justus Masa, Dulce M. Morales, Ondrej Tomanec, Vaclav Ranc, Martin Petr, Patrick Wilde, Yen‐Ting Chen, Radek Zboril, Wolfgang Schuhmann, Roland A. Fischer

**Affiliations:** ^1^ Chair of Inorganic and Metal–Organic Chemistry Department of Chemistry and Catalysis Research Centre Technical University of Munich 85748 Garching Germany; ^2^ Regional Centre of Advanced Technologies and Materials Faculty of Science Palacky University Šlechtitelu˚ 27 783 71 Olomouc Czech Republic; ^3^ Analytical Chemistry Center for Electrochemical Sciences (CES) Faculty of Chemistry and Biochemistry Ruhr‐University Bochum 44870 Bochum Germany; ^4^ Center of Molecular Spectroscopy and Simulation of Solvent‐driven Processes (ZEMOS) Ruhr‐University Bochum Universitätsstr. 150 44801 Bochum Germany

**Keywords:** electrocatalysis, liquid‐phase exfoliation, mechanochemical synthesis, oxygen evolution, 2D materials, zeolite imidazole frameworks (ZIFs)

## Abstract

2D layered materials, including metal‐di‐chalcogenides and transition metal layered double hydroxides, among others, are intensively studied because of new properties that emerge from their 2D confinement, which are attractive for advanced applications. Herein, 2D cobalt ion (Co^2+^) and benzimidazole (bIm) based zeolite‐imidazole framework nanosheets, ZIF‐9(III), are reported as exceptionally efficient electrocatalysts for the oxygen evolution reaction (OER). Specifically, liquid‐phase ultrasonication is applied to exfoliate a [Co_4_(bIm)_16_] zeolite‐imidazole framework (ZIF), named as ZIF‐9(III) phase, into nanoscale sheets. ZIF‐9(III) is selectively prepared through simple mechanical grinding of cobalt nitrate and benzimidazole in the presence of a small amount of ethanol. The resultant exfoliated nanosheets exhibit significantly higher OER activity in alkaline conditions than the corresponding bulk phases ZIF‐9 and ZIF‐9(III). The electrochemical and physicochemical characterization data support the assignment of the OER activity of the exfoliated nanosheet derived material to nitrogen coordinated cobalt oxyhydroxide N_4_CoOOH sites, following a mechanism known for Co‐porphyrin and related systems. Thus, exfoliated 2D nanosheets hold promise as potential alternatives to commercial noble metal electrocatalysts for the OER.

The development of efficient technologies for water splitting to produce hydrogen as an energy carrier is considered as the most promising energy conversion technology for the future.^1^, ^2^, ^3^, ^4^ The oxygen evolution reaction (OER) is a key reaction in water splitting, which is slow and thus energetically inefficient. Some metal oxides are highly active and durable electrocatalysts for OER, among which IrO_2_ and RuO_2_ are regarded to be the benchmark OER catalysts in both acidic and alkaline solutions.^5^ However, the high cost and scarcity of these noble elements are obstacles to their widespread commercial use. To this end, hydrogen production by electrochemical water splitting is still not wide spread as a cost‐effective technology for energy conversion.^6^ Therefore, it is desirable to discover and develop efficient alternative electrocatalysts, in particular for OER, based on inexpensive, earth abundant and environmentally benign elements and materials.

2D layered materials like dichalcogenides and double layer hydroxides have recently been demonstrated to be among the most attractive OER candidates in terms of both catalytic activity and potential stability.^7^, ^8^, ^9^, ^10^ This development motivated the search for new advanced OER electrocatalysts based on alternative 2D layered materials. A promising class of novel systems are 2D inorganic–organic hybrid materials derived from crystalline coordination networks, in particular from metal–organic frameworks (MOFs).^11^, ^12^, ^13^, ^14^, ^15^ In general, MOFs are based on the self‐assembly of metal ions and organic linkers to yield 3D crystalline porous coordination networks. MOFs may feature highly exposed active sites on their surface, including coordinatively unsaturated metal sites suited as catalytic reaction centers.^16^ Thus, 2D MOFs offer humongous possibilities and a wide parameter space, allowing for eventually achieving superior and unusual material properties that cannot be obtained otherwise. 2D MOFs exhibit a layered structure with strong in‐plane coordination bonds and weak interactions between the layers (e.g., van der Waals forces and hydrogen bonding). This property may provide favorable interaction between active sites and substrate molecules with a smaller diffusion barrier as compared to their 3D bulk MOF counter parts.^16^, ^17^, ^18^, ^19^, ^20^, ^21^ Recently, Zhao and co‐workers reported 2D MOF nanosheets derived from a Cu and TCPP (tetrakis(4‐carboxyphenyl)porphyrin). The authors demonstrated remarkable fluorescent sensing performance of the material and the capability for simultaneous detection of multiple DNA targets.^21^ Tang and co‐workers obtained Ni–Co MOF nanosheets through a solvothermal process and exploited them for OER.^22^

Here, we report an unprecedented facile and scalable strategy to obtain cobalt zeolite imidazole framework 2D layers by liquid exfoliation of the ZIF‐9(III) phase (**Figure** **1**a). The material derived by exfoliation exhibited highly efficient OER activity in alkaline media. The bulk layered cobalt‐ZIF‐9(III) phase was synthesized by simple mechanochemical grinding of cobalt nitrate and benzimidazole (bIm) as organic linker in the presence of ethanol. The exfoliation leads to a material with the same chemical composition of bulk ZIF‐9(III) but exhibiting greatly enhanced OER activity, which is attributed to increased accessibility of high density redox active cobalt oxyhydroxide active sites with a nitrogen coordination environment (N_4_CoOOH).

**Figure 1:**
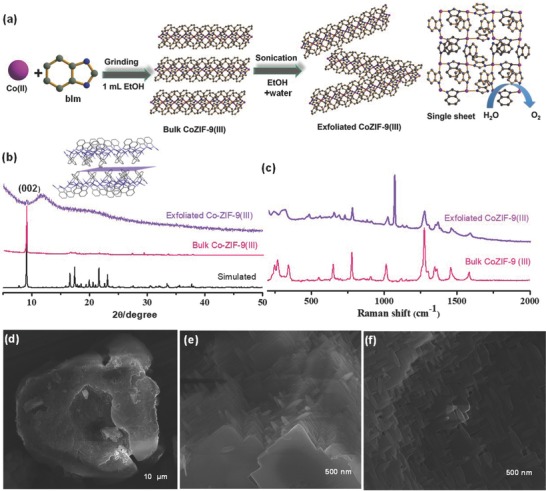
a) Schematic representation of 2D Co‐ZIF‐9(III) nanosheets exfoliated in liquid phase from bulk Co‐ZIF‐9(III). The bulk phase was prepared by grinding a cobalt salt with bIm linkers constituting 2D nanosheets, which stack along the crystallographic *c* direction via weak van der Waals interactions. b) Powder XRD patterns of simulated, bulk and exfoliated Co‐ZIF‐9(III) with the inset showing the arrangement of the Co‐ZIF‐9(III)(in case of exfoliated material, the broad peak at 13° corresponds to silicon grease), where hydrophobic benzimidazole groups are separated by the (002) plane along crystallographic *a*‐axis. c) Raman spectroscopy of bulk and exfoliated Co‐ZIF‐9(III) showing structure rigidity. d–f) FESEM images of bulk Co‐ZIF‐9(III) showing bundles of nanoscale plates.

Zeolitic imidazole frameworks (ZIFs) represent an important subgroup of chemically highly robust MOFs, especially in alkaline solution.^23^ The materials are named after the resemblance of the Zn–imidazole–Zn linkage to the Si–O–Si linkage found in zeolites. ZIFs often feature the same structural topologies as zeolites. To the best of our knowledge, ZIF‐7, ZIF‐9, and EMM‐19 are the only ZIFs known to exhibit a phase transition from a nearly nonporous to a porous structure upon adsorption of guest molecules.^23^ There are three related ZIF‐9 phases. The two phases “ZIF‐9(I)” (Co(bIm)_2_, cubic system) and “ZIF‐9(II)” (Co_9_(bIm)_18_ triclinic system) were obtained by heating ZIF‐9‐I. The permanently nonporous third phase “ZIF‐9(III)” was produced by a slurry aging experiment after leaving ZIF‐9‐II in water at room temperature for one week. Most importantly, the dense coordination network structure of ZIF‐9(III) features a (4,4) square planar grid layer structure formed by fourfold linked corner‐shared Co(II) benzimidazole tetrahedra as Co_4_(bIm)_16_ (Figure S1, Supporting Information)_._ The 2D layers stack along the crystallographic c direction and are interconnected by weak van der Waals interactions (Figure 1a and Figure S2, Supporting Information).

We demonstrate herein that very simple and easy scalable mechanochemical synthesis by grinding of the reactants cobalt nitrate, benzimdazole and sodiumbicarbonate in the presence of ethanol (≈1 mL) for 2 min selectively yields bulk Co‐ZIF‐9(III) (for complete details, see the Experimental Section in the Supporting Information). As revealed by field emission scanning electron microscopy (FESEM), the as‐synthesized grinded product consists of multiple, closely packed bundles of crystalline plates of micrometric thickness (Figure 1d–f and Figure S3, Supporting Information). Transmission electron microscopy (TEM) confirms the existence of stacked nanosheets (Figure S4, Supporting Information). The powder X‐ray diifraction (XRD) pattern exhibits sharp lines without a shift in the peak positions with respect to the simulated XRD pattern (Figure 1b and Figure S5, Supporting Information). Structural refinements and comparison with Co‐ZIF‐9(III) reference crystal parameters were performed using the Pawley fitting (Figure S6, Supporting Information). The lattice parameters obtained from the Pawley fit agree well with lattice parameters from single crystal X‐ray diffraction.

The interlayer space of Co‐ZIF‐9(III) is occupied by the hydrophobic benzimidazole groups and the layers are held together only by weak van der Waals interactions (Figure 1a). Thus, it is expected that liquid‐phase exfoliation of Co‐ZIF‐9(III) can be achieved similarly as in the case of graphite for graphene. Co‐ZIF‐9(III) powder was treated with ultrasound in ethanol (0.15 mg mL^−1^) for 20 min to obtain a milky colloidal suspension comprising a mixture of fully exfoliated and partially exfoliated nanosheets (Figure S7, Supporting Information). The obtained colloidal solution exhibited the Tyndall effect (Figure S8, Supporting Information). Extending the sonication time in the presence of ethanol as dispersant solvent yields a higher degree of exfoliation. Powder XRD of the collected exfoliated material shows only one broad peak, which can be indexed to the (002) planes of ZIF‐9(III) (Figure 1b). The absence of the other Bragg diffraction peaks (as expected for the properly ordered bulk material) indicate the exfoliation into randomly oriented layers.^21^ The intact coordination network within the Co‐ZIF‐9(III) layers after exfoliation was confirmed by Raman and Fourier transform infrared (FT‐IR) spectroscopy. All the bands were found in the same position as for bulk ZIF‐9(III) (Figure 1c and Figure S9, Supporting Information). The colloidal solution of the nanosheets was dispersed onto a flat substrate for characterization using TEM and atomic force microscopy (AFM). The AFM images reveal structures with typical lateral dimensions of 40–80 nm and thickness of about 2–4 nm (**Figure** **2**d and Figure S10, Supporting Information). The transmission electron microscope (TEM) images confirm the existence of randomly oriented stacked ultra‐thin layers (Figure 2a–c). Selected area electron diffraction (SAED) from exfoliated Co‐ZIF‐9(III) nanosheets confirmed the single crystalline behavior of those regions. The data indicate the in‐plane retention of the crystallographic ordering of the pristine bulk material also for the nanosheets (Figure S11, Supporting Information).^24^ A typical HAADF‐TEM image of the exfoliated Co‐ZIF‐9(III) material shows a uniform distribution of C, N, and Co in the whole nanosheet (Figure 2e–h). Textural parameters such as surface area and pore size distribution of the collected exfoliated Co‐ZIF‐9(III) powder material were obtained from N_2_‐adsorption–desorption isotherm measurements. The BET surface area is around 19 m^2^ g^−1^ with a pore size distribution of 2–15 nm (calculated from the non‐local density functional theory (NLDFT) method; Figure S12, left, Supporting Information).

**Figure 2:**
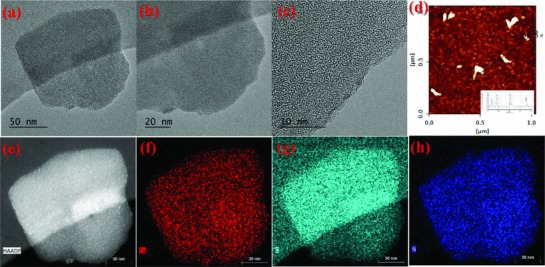
a–c) HAADF‐TEM Image of liquid exfoliated 2D ZIF‐9(III) showing 2D ultrathin nanosheets; d) AFM image shows thickness of nanosheets around 2–4 nm. e–h) Elemental analysis confirms the composition, where cobalt, nitrogen and carbon elements are homogeneously distributed throughout the sample.

2D Co‐ZIF‐9(III) nanosheets are expected to be superior in catalytic performance as compared to the corresponding bulk phase materials due to a high active site density arising from enhanced accessibility of the active sites with a smaller diffusion barrier for substrate molecules. As a proof‐of‐concept, the electrocatalytic performance of the obtained 2D exfoliated Co‐ZIF‐9(III) nanosheets towards the oxygen evolution reaction (OER) was studied in alkaline conditions (1.0 m KOH) along with its bulk phases. A suspension of the catalyst (5.0 mg mL^−1^) dispersed in a water/ethanol mixture containing 0.2 vol% Nafion was drop cast on a finely polished glassy carbon electrode to form a film with a catalyst loading of 210 µg cm^−2^. The material underwent irreversible electrochemical transformation upon potential application as depicted by the changes that take place during potential cycling between 0.9 and 1.6 V vs RHE (**Figure** **3**a, inset). During the first scan, two oxidation processes with a weak peak at ≈1.2 V, and a more pronounced peak at ≈1.35 V can be discerned, ascribed to the oxidation of Co^2+^ to Co^3+^ and Co^4+^, respectively.^25^ In subsequent CVs, the material undergoes reversible redox transitions, Co^2+^ ↔ Co^3+^ + e, which occur at about 1.2 V. These reversible peaks initially increased in intensity with potential cycling before becoming stable after about 50 CVs. The increase in intensity of the Co^2+^ ↔ Co^3+^ + e redox peaks indicates the formation of various cobalt species such as oxo, hydroxo, and hydroperoxo at the nitrogen coordinated cobalt sites of the Co‐ZIF‐9(III) nanosheets with potential cycling. Specifically, under oxidizing conditions, the Co^2+^ sites are stepwise transformed to Co^3+^, which is most likely coordinated to a hydroperoxide moiety ([OOH]^−^). Such kind of species are the expected form of the catalytic active site prior to oxygen evolution. A noteworthy feature of the Co^2+^ ↔ Co^3+^ + e redox processes is their high reversibility, which is important for fast kinetics of reactions involving a redox catalytic mechanism (see Figure 5 for proposed mechanism). Such high reversibility of the Co^2+^ ↔ Co^3+^ + e redox processes is untypical of purely inorganic cobalt based catalysts (e.g., cobalt oxides) and thus a clear indication that the four coordination nitrogen atoms of the imidazole ligands strongly mediate in the Co^2+^ ↔ Co^3+^ + e redox processes. The OER activity of the exfoliated Co‐ZIF‐9(III) nanosheets in 1.0 m KOH is shown in Figure 3a as linear sweep voltammograms (LSVs), recorded at a scan rate of 10 mV s^−1^ and electrode rotation at 1600 rpm. The results are presented together with the OER data of bulk phases of ZIF‐9(I) and ZIF‐9(III). The comparison demonstrates superior OER activity of the 2D nanosheets in relation to the bulk materials. The 2D nanosheets required 1.61 V (0.38 overpotential (η)) to drive a current density of 10 mA cm^−2^ whereas bulk Co‐ZIF‐9‐I and Co‐ZIF‐9(III) required 1.65 V (η = 0.42 V) and 1.65 V (η = 0.43 V), respectively, to drive the same current density. The Tafel slope $\left(\frac{\partial E}{\partial (\ln i)}=\frac{-2.303RT}{\alpha F}\right)$ of the OER was 55, 57, and 56 mV dec^−1^, respectively, on the exfoliated Co‐ZIF‐9(III) 2D sheets, bulk phases of Co‐ZIF‐9(I) and Co‐ZIF‐9(III). The Tafel slopes are close to the theoretical value of $\frac{-2.303RT}{\alpha F}$or 59 mV, where the first electron transfer step is rate limiting and the coverage with adsorbed species is low. The similarity of the Tafel slopes indicates that the mechanism of the OER was similar on all the catalysts and further, that the active sites are most certainly the same. The exceptionally high OER activity of the 2D Co‐ZIF‐9(III) nanosheets compared to its bulk counterparts must therefore arise from its comparatively much higher active site density due to a higher degree of exposure of the edge sites in addition to the basal sites. The performance of the exfoliated 2D Co‐ZIF‐9(III) nanosheets, expressed by average overpotential at a current density of 10 mA cm^−2^, was further compared against that of selected cobalt based OER catalysts as well as IrO_2_ and RuO_2_ (Figure 3c and **Table** **1**).

**Figure 3:**
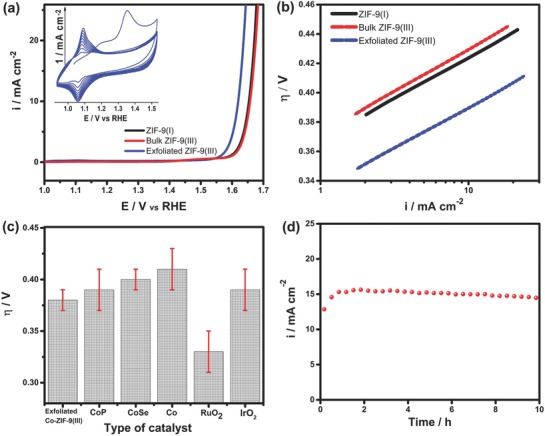
a) Linear sweep voltammograms showing the OER activity of exfoliated Co‐ZIF‐9(III) 2D nanosheets in comparison with bulk phases of ZIF‐9‐I, ZIF‐9‐III. The inset of (a) shows cyclic voltammograms of exfoliated Co‐ZIF‐9(III) nanosheets in 1.0 m KOH recorded at a scan rate of 100 mV s^−1^. b) Comparison of the Tafel plots of the exfoliated ZIF‐9(III), bulk ZIF‐9(III) and ZIF‐9(I). c) The average OER activity of exfoliated Co‐ZIF‐9(III) expressed as the overpotential at a current density of 10 mA cm^−2^ compared with that of selected cobalt based catalysts (Co, CoP, and CoSe), and IrO_2_ and RuO_2_, using data taken from the references provide in Table 1; and d) chronoamperometric stability data at 1.7 V versus RHE of the 2D ZIF‐9‐III nanosheets recorded in 1.0 m KOH with electrode rotation at 1600 rpm.

**Table 1:** Comparison of the OER performance of 2D Co‐ZIF‐9(III) nanosheets with the activities of selected nonprecious metal catalysts from the literature. CoP, cobalt phosphide; NF, nickel foam; MWCNT, multiwalled carbon nanotubes

Material/substrate	Loading [mg cm^−2^]	Electrolyte	η [V] 10 mA cm^−2^	Tafel slope [mV dec^−1^]	Ref.
2D CoZIF‐9(III) sheets	0.21	1.0 m KOH	0.38	55	This work
CoP/NF	6.20	1.0 m KOH	0.39	65	26
Co_3_O_4_/GC	1.00	1.0 m KOH	0.53	47	27
CoP	0.2	1.0 m KOH	0.39	50	28
CoP/N‐MWCNT	–	0.1 m NaOH	0.33	50	29
RuO_2_	–	0.1 m KOH	0.33		5
IrO_2_	–	0.1 m KOH	0.39	89	5
	–		Activity		
Ni:Pi‐Fe	–	1.0 m KOH	0.290 V at 500 mA cm^−2^	–	32
		5.0 m KOH	0.332 V at 1600 mA cm^−2^		
NiCoP/NF	–	1.0 m KOH	0.308 V at 50 mA cm^−2^	–	33
Co/Co_2_P/NF	0.22	1.0 m KOH	0.190 V at 50 mA cm^−2^	–	34

The electrochemical properties of the catalyst were characterized by impedance spectroscopy (EIS). The resistances to interfacial charge transfer across the electrocatalytic films derived from bulk Co‐ZIF‐9(I), bulk Co‐ZIF‐9(III), and exfoliated 2D Co‐ZIF‐9(III) nanosheets were determined from the diameter of the semicircles in the corresponding Nyquists plots (Figure S13, Supporting Information). The resistances decreased in the order Co‐ZIF‐9(I) (40.7 Ω) > bulk Co‐ZIF‐9(III) (23.0 Ω) > exfoliated 2D Co‐ZIF‐9(III) nanosheets (≈0.0 Ω). This sequence indicates inherently higher electronic conductivity of the films derived from the exfoliated material as compared to the bulk materials, which partly accounts for the superior OER activity of the 2D Co‐ZIF‐9(III) nanosheet‐derived electrode coating.

The average OER performance of the exfoliated 2D Co‐ZIF‐9(III) nanosheets based on three replicate measurements is superior to the performance reported for most cobalt‐based catalysts on a passive support.^26^, ^27^, ^28^, ^29^, ^30^ The OER performance of the 2D nanosheets was compared against benchmark cobalt based catalysts, and other nonprecious catalysts reported in the literature as summarized in Table 1.^30^, ^31^, ^32^, ^33^, ^34^, ^35^, ^36^, ^37^, ^38^ The turnover frequency (TOF) of the 2D exfoliated nanosheets at 1.65 V was 0.2 s^−1^ (mole Co)^−1^ (calculated on the basis of the 11.1% total amount of cobalt in the catalyst, see the Experimental Section). This value is one order of magnitude higher than that for Co_3_O_4_ (0.04 s^−1^) at the same conditions. The TOF essentially depicts the inherent activity of the active sites for a given catalyst and the results indicate that the cobalt sites in the Co‐ZIF‐9(III) nanosheets are potently more active than the cobalt sites in Co_3_O_4_. This observation is attributed to the 2D confinement of the cobalt centers in the ZIF‐nanosheets and their synergistic interaction with nitrogen, as well as fast mass transport of reactants and products owing to the unique morphology of the catalyst.

The long‐term performance of the exfoliated 2D Co‐ZIF‐9(III) nanosheets was investigated by a sequence of chronoamperometric measurements at 1.7 V versus RHE for 900 s followed by LSV activity measurements, repeating the protocol for a total duration of 10 h. Figure 3d shows the average current densities recorded as function of time. During the first hour, the current density increased from 12.6 to 15.6 mA cm^−2^, then decreased gradually afterward. Nonetheless, despite the observed activity, the average current density after 10 h remained higher than the initial value. The initial increase of the current density is due to activation of the catalyst and concomitant increase in accessibility of the active sites. Physical detachment of the catalyst films was visible and it is certainly one of the major causes of the decline in activity following the initial activation.

We probed the structure of the material derived from exfoliated 2D Co‐ZIF‐9(III) nanosheets under OER conditions by coupled in situ Raman spectroscopy, to get insight into the active state of the catalyst during OER. **Figure** **4**a shows Raman spectra recorded at different electrode potentials in KOH solution at pH 12. At open circuit potential (OCP), i.e., without an externally applied potential, the most imminent Raman bands were at 465.4, 521.3, 548.1, 642, and 778.5 cm^−1^. The band at ≈521.3 cm^−1^ is assigned to Co(OH)_2_ species and is due to ν(Co–O) (A_g_) symmetric stretching mode, while that at ≈548.1 cm^−1^ is due to ν(Co–N) stretching mode.^39^, ^40^, ^41^ Upon application of an oxidizing potential, the bands at 465.4 cm^−1^ and at 548.1 cm^−1^ diminish in intensity while the one at 523 cm^−1^ is accentuated. Meanwhile, the band at 778.5 cm^−1^, ascribed to deformation of the complex, also decreases in intensity with increase of the applied potential. The band initially centered at 642 cm^−1^ at OCP, becomes less sharp and its center moves to a slightly lower Raman shift. These modifications were irreversible. The original Raman bands were not regenerated upon reversal of the applied potential in similar steps. Thus, under the influence of the applied potential, the exfoliated 2D Co‐ZIF‐9(III) nanosheets underwent irreversible transformation due to formation of pendant hydroxide and oxyhydroxide groups at the uncoordinated Co sites of the nanosheets. In the following we denote these sites as N_4_CoX (X = hydroxo, oxo, hydroperoxo, peroxo, etc.) in order to emphasize the particular molecular N‐donor coordination environment being similar to the Co‐ZIF‐9(III) base material, however in contrast to CoX species being present at inorganic Co oxide surfaces. A survey of the literature did not disclose any published data on the Raman spectral features of N_4_CoOOH type species under alkaline conditions as a reference for comparison with our spectroscopic studies. The Raman spectral features of CoOOH surface species at inorganic cobalt oxides, which is most closely related to the proposed molecular N_4_CoOOH species, was thus used as the reference for comparison and basis for discussion. Note, the notation “CoOOH” refers to oxo and hydroxo species being simultaneously present at the Co‐oxide surface. The most intense Raman band of CoOOH species is expected at about 503 cm^−1^ with additional less intense peaks at about 641, 572, and 705 cm^−1^, on the basis of Raman studies on a reference CoOOH material by Yang et al.^42^ and Masikhwa et al.^43^ Meanwhile, Co(OH)_2_ species exhibits its most intense Raman peak at about 523 cm^−1^. Owing to evidence of the prevalence of the Co–N coordination moieties, we therefore attribute the broad band stretching from about 500 to 570 cm^−1^, whose intensity increased with applied potential, to be a convolution of N_4_CoO (Co^4+^), N_4_CoOH (Co^3+^), and probably also N_4_CoOOH (Co^3+^)_._ The proposed formation of such kind of coordinated “N_4_CoOOH” species in the exfoliated Co‐ZIF‐9(III) nanosheets as equivalent to the inorganic “CoOOH” species under OER conditions is consistent with the expected preoxidation steps that proceed the evolution of oxygen at inorganic metal/metaloxide active sites,^44^, ^45^ involving the reaction sequence: i) M + OH^−^ → MOH + e^−^; ii) MOH + OH^−^ → MO + H_2_O + e^−^; iii) MO + OH^−^ → MOOH; iv) MOOH + OH^−^ → MO_2_ + H_2_O + e^−^, and v) MO_2_ → M + O_2_ + e^−^ . We transfer this mechanism to our case. Thus, equations (i)–(iii) refer to the formation of oxo, hydroxo, and possibly hydroperoxo groups at the Co centers exposed on the exfoliated 2D ZIF‐9(III) sheets, where in (i) and (ii) the cobalt site is stepwise oxidized from the Co^2+^ to the Co^4+^ state, in (iii), the proposed N_4_CoOOH hydroperoxo species (Co^3+^) is formed and in steps (iv) and (v) O_2_ isoxidatively released and the N_4_Co^2+^ active site is finally regenerated to repeat the cycle (**Figure** **5**). The Tafel slope of 55 mV dec^−1^, for the OER on the exfoliated 2D nanosheet indicate that the first electron transfer step is the rate‐determining step. Since in the ZIF‐9(III) nanosheets Co is present as Co^2+^, reaction (iii) is identified as the rate‐determining step, yielding excellent agreement between the electrochemical data and in situ Raman spectroscopy observation. Importantly, the N_4_Co coordination between the cobalt ions and the benzimidazole ligand remains intact during the OER as confirmed through operando Raman imaging of the Co–N band (Figure 4i), and in the aftermath of long‐term electrochemical testing. Post‐mortem XRD analysis of Co‐ZIF‐9(III) after 3 h of constant polarization at 10 mA cm^−2^ (Figure S14, Supporting Information) exhibits no peaks corresponding to ZIF‐9(III) because of randomly organized nanosheet and confirms no presence of oxide based impurities. The post‐mortem Raman spectrum shows an absorption band positioned at 548 cm^−1^ assigned to Co–N. Figure 4b (inset) shows results of cluster analysis, where the blue regions represents a cluster rich with Co—N bonds. Further, Raman spectroscopy confirms the structure of the catalyst remained intact even after electrocatalytic measurements.

**Figure 4:**
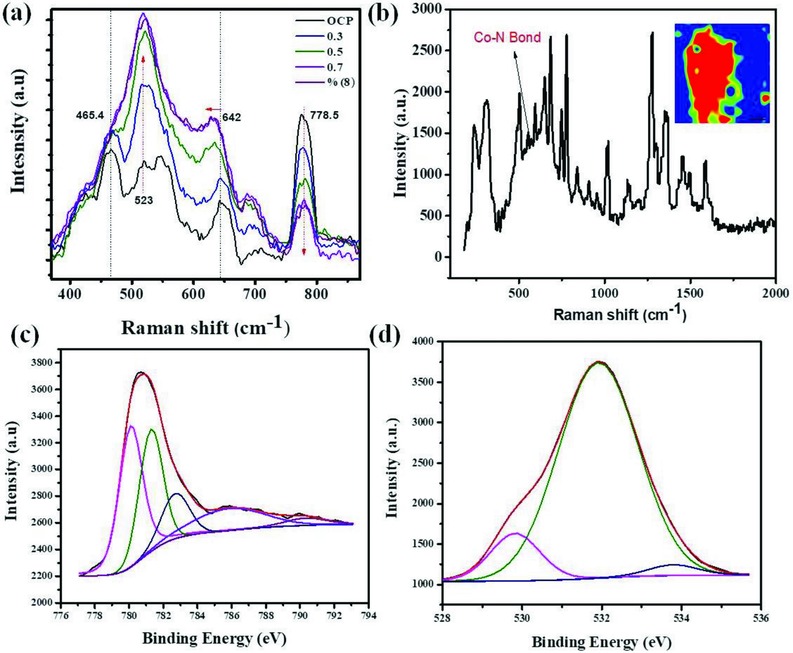
a) In situ Raman spectra acquired at different electrode potentials (E vs Ag/AgCl 3 m KCl) of exfoliated Co‐ZIF‐9(III) nanosheets in KOH (pH 12); b) post‐mortem Raman spectrum. The band at 548 cm^−1^ is assigned to the Co—N bond, and mapping (inset) of the sample where the blue color represents a higher abundance of Co—N bonds deconvoluted high‐resolution XPS spectra of c) the Co 2p region and d) the O 1s region.

**Figure 5:**
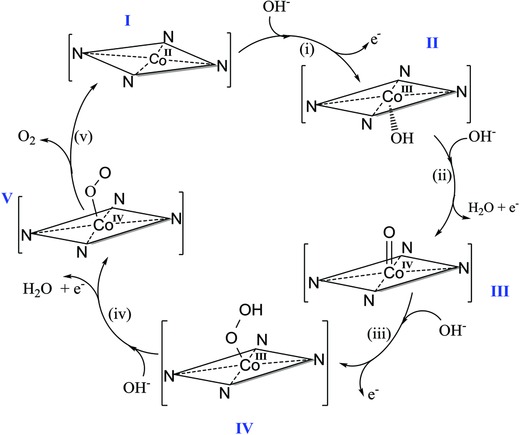
Proposed scheme of the nature of the active site of the exfoliated Co‐ZIF‐9(III) 2D catalyst and the underlying mechanism of the oxygen evolution reaction (OER).

We analyzed this sample by XPS to elucidate the nature of the coordination environment. The survey spectrum shows the presence of cobalt, oxygen, carbon and nitrogen, and their corresponding composition in atomic percentage (C 1s (70.0%), O 1s (18.8%), Si 2p (4.1%), Co 2p (3.9%), and N 1s (3.2%) (Figure S15, Supporting Information). The O 1s peak was deconvoluted into three components at 529.82, 531.91, and 533.79 eV corresponding to N_4_CoOOH, O=C and O—C, respectively (Figure 4d). The N 1s peak was deconvoluted into two contributions at 398.97 and 400.45 eV characteristic of pyridinic and pyrrolic nitrogen, respectively (Figure S16, Supporting Information). The high‐resolution spectrum of Co 2p with the spectral components at 780.10, 781.28, 782.70 eV, and satellites at 785.82 and 790.31 eV (Figure 4c) corresponds to N_4_CoOOH.^42^ The features and binding energies of the deconvoluted core Co 2p spectrum agree fairly well with those of the related CoOOH species, where the core Co 2p spectrum of CoOOH was fitted with Co 2p3/2 peaks at 780.40 and 781.7 eV with corresponding satellites at 783.8 and 789.9 eV respectively.^42^ Additionally, the deconvoluted O 1s spectral components at 529.0, 532.5, and 534.6 eV (Figure 4d), are ascribed to O from the oxide and hydroxide of N_4_CoOOH, respectively, similar to CoOOH.^42^, ^46^

Post‐mortem TEM images show a layer morphology and elemental mapping by electron energy loss spectroscopy (EELS) also confirms the presence of cobalt, carbon, nitrogen, and oxygen distributed throughout the sample (Figure S17, Supporting Information). All these characterizations indicate that the various oxygen species coordinated to the Co^3+/4+^ sites of the Co‐ZIF‐9(III) nanosheets does not significantly alter the intrinsic, overall crystallographic features of the material. Therefore, besides the high active site density of the 2D nanosheets, the N atoms coordinated to Co are obviously expected to influence the electronic properties of the Co sites, and thus of the various intermediates, leading to the excellent OER activity of the exfoliated 2D ZIF‐9(III) nanosheet derived catalyst, and subsequently, to the observed enhancement of the OER activity.

For a better understanding of the data assignment and our reasoning based on the reversible formation of N_4_CoOOH as the key species, a schematic representation of a conceptual mechanism for activation of OH^−^ ions at the Co‐ZIF‐9(III) 2D nanosheets is illustrated in Figure 5. In this scheme, activation of the OH^−^ ions is initiated by their chemisorption (i.e., coordination) via step i) at the initially tetra‐N‐coordinated Co^2+^ site (structure I). Due to the structural flexibility of the 2D MOF nanosheets, a local transformation to a pseudo square‐planer pyramidal coordination environment of the cobalt center is suggested (structure II). Note that it is also possible to coordinate two hydroxyl units to obtain a (*trans*) Co(OH)_2_ species with an octahedral cobalt center (which is not shown in the Figure for clarity). Accordingly, during step i) this cobalt site is electrochemically oxidized to Co^3+^, hydroxylated and still coordinated to four nitrogen atoms. Thus, the OH^−^ (hydroxo), O^2−^ (oxo), OOH^−^ (hydroperoxo), and O_2_^2−^ (peroxo) attachment and transformations all occur at the axial positions of cobalt sites (structures II–V) of different oxidation states as depicted in the steps (i)–(iv). Finally, electrochemical oxidation of the structures IV and V liberates O_2_ (by formal reductive elimination from the peroxide structure) and regenerates the N_4_Co^2+^ center in structure I. It should be noted that such a situation of the coordinatively flexible Co‐ZIF‐9(III) nanosheets, with Co^2+/3+/4+^ coordinated to four benzimdazole groups efficiently mimics the active site structure of porphyrin based cobalt complexes commonly employed as homogeneous water oxidation catalysts.^47^, ^48^, ^49^, ^50^

In summary, the unique physical and chemical properties of 2D materials, possessing large lateral dimensions and nanometric thickness, makes them ideal for various applications in energy conversion. 2D cobalt‐based ZIF‐9(III) nanosheets synthesized by simple and facile mechanochemical grinding with subsequent ultrasonication assisted liquid‐phase exfoliation, exhibited excellent OER electrocatalytic performance. The electrocatalytic activity of the 2D Co‐ZIF‐9(III) nanosheet derived electrocatalyst coating in 1.0 m KOH is significantly higher in terms of current density and overpotential compared to the bulk Co‐ZIF‐9 counterparts. The exfoliated 2D Co‐ZIF‐9(III) derived electrocatalyst obviously exhibits the advantage of more accessible active sites as compared to 3D bulk MOF crystals. This work therefore opens up new avenues for efficient production of electrocatalytic materials based on or derived from exfoliated 2D metal–organic frameworks or 2D coordination polymers.

## Conflict of Interest

The authors declare no conflict of interest.

## Supporting information

Supplementary
