# Supplementary material for: Ultrathin 2D Cobalt Zeolite‐Imidazole Framework Nanosheets for Electrocatalytic Oxygen Evolution
Source: Adv Sci (Weinh). 2018 Oct 13;5(11):1801029. doi: 10.1002/advs.201801029 (PMC6247023; doi:10.1002/advs.201801029)
Supplement: Supplementary file 1 — Supplementary [file ADVS-5-1801029-s001.pdf]

Copyright WILEY-VCH Verlag GmbH & Co. KGaA, 69469 Weinheim, Germany, 2018.

ADVANCED SCIENCE Open Access

Supporting Information

for *Adv. Sci.,* DOI: 10.1002/advs.201801029

Ultrathin 2D Cobalt Zeolite-Imidazole Framework Nanosheets for Electrocatalytic Oxygen Evolution

Kolleboyina Jayaramulu, Justus Masa,* Dulce M. Morales, Ondrej Tomanec, Vaclav Ranc, Martin Petr, Patrick Wilde, Yen-Ting Chen, Radek Zboril, Wolfgang Schuhmann,* and Roland A. Fischer**

WILEY-VCH

Copyright WILEY-VCH Verlag GmbH & Co. KGaA, 69469 Weinheim, Germany, 2016.

Supporting Information

Ultrathin Two-Dimensional Cobalt Zeolite-Imidazole Framework Nanosheets for Electrocatalytic Oxygen Evolution

Kolleboyina Jayaramulu,* Justus Masa,* Dulce M. Morales, Ondrej Tomanec, Vaclav Ranc, Martin Petr, Patrick Wilde, Yen-Ting Chen Radek Zboril, Wolfgang Schuhmann* and Roland A. Fischer*

Experimental Section

Materials

All reagents and solvents were commercially available and used as supplied without further purification. $Co(NO_3)_2·6\ H_2O$, benzimidazole, sodiumbicarbonate were obtained from Sigma-Aldrich.

Synthesis of Co-ZIF-9/Co-ZIF-9(I)

The synthesis procedure of Co-ZIF-9 has been described by Park et al.[23] $Co(NO_3)_2•6H_2O$ (0.210 g) and benzimidazole (0.060 g) were mixed in a 20 mL glass vial and dissolved by addition of DMF (25 mL). Later, the glass vial was tightly closed and placed in an oven at 130 °C for 2 days. After cooling to room temperature, the supernatant was decanted and the blue/purple crystals were washed three times with DMF and three times with methanol. Methanol was decanted and replaced once per day during the course of three days and later it was removed under vacuum. The product was heated under vacuum to 250 °C for 5 h. The sample was cooled to room temperature and stored under ambient conditions.

Synthesis of bulk Co-ZIF-9 (III)

Mechanical synthesis: All chemicals employed were commercially available (Sigma-Aldrich and Acros Organics) with purities of 98 % or above. $Co(NO_3)_2•6H_2O$ (88.50 mg, 0.30 mmol), bIm (71.45 mg, 0.60 mmol) and sodium bicarbonate ($NaHCO_3$, 47.38 mg, 0.56 mmol) were mixed in a mortar presence of 2 ml ethanol, grinding them approx. 10 minutes. The product was washed with water to get rid of remaining $NaHCO_3$ and $NaNO_3$. A blue-pink powder product was obtained for characterization.

Liquid-phase exfoliation of Co-ZIF-9 (III): 15 mg of the bulk Co-ZIF-9(III) was added to 10 mL of dispersion solvent (water/ethanol 1:1 by volume). The mixture was ultrasonicated for 5 h (BRANSON 5510-MTH). The resulting dispersion was centrifuged at 4000 rpm for 10 min to remove precipitates.

Preparation of electrodes

For evaluation of activity, glassy carbon electrodes of 3.8 mm diameter (A = 0.1134 cm^2) were polished with 0.05 µm Al_2O_3 paste and rinsed with water and ethanol. The catalyst inks were prepared by adding 5 mg mL^{-1} active material to a solution consisting of 49 vol% water, 49 vol% ethanol and 2 vol% Nafion solution. After sonicating the mixture for 10 min, 5.3 µL of the ink were drop-cast onto the glassy carbon electrodes and dried at room temperature. For the stability tests graphite electrodes of 5 mm diameter (A = 0.1964 cm^2) modified with 8.3 µL of the catalyst ink were used as working electrodes.

Investigation of electrochemical activity

Electrochemical measurements were controlled with an Autolab PGSTAT30 (Metrohm) potentiostat, equipped with a Metrohm rotator (RDE 80793) and motor control unit. The experiments were conducted using a glass cell in a three-electrode configuration, where the

glassy carbon electrode modified with the catalyst was used as working electrode (WE), a platinum wire was used as counter electrode (CE), a home-made Ag/AgCl (3 M KCl) electrode as reference (RE) and 1.0 M KOH solution as supporting electrolyte. The OER performance was investigated by means of linear sweep voltammetry (LSV) in the potential range from 0.9 to 2.0 V vs. RHE, at a scan rate of 10 mV s^{-1} and with a rotation speed of the WE of 1600 rpm. The data was collected after reaching a constant response from cyclic voltammograms in the range from 0 to 1.5 V at a scan rate of 100 mV s^{-1}. Electrochemical impedance spectroscopy was used to determine the resistance of the electrolyte, measured from 50 kHz to 10 Hz at the open circuit potential with AC perturbation of 10 mV. All potentials were converted to the RHE and IR-drop compensated according to the following expression: $E_{RHE} = E_{Ag/AgCl} + 0.207 + 0.059\ pH - iR$

The turn-over frequency (TOF) of the catalysts during the OER was determined at 1.65 V using the equation, TOF = i(A)/4eN, where N is the estimated number of moles of cobalt atoms that participate in the reaction, e the electronic charge, and i the current, and assuming that all the atoms loaded on the electrode participate in the reaction.

Characterization

The synthesized materials were characterized by different techniques. X-ray diffraction (XRD) data of all samples were collected by the X'Pert PRO PANanalytical equipment (Bragg-Brentano geometry with automatic divergence slits, position sensitive detector, continuous mode, room temperature, Cu-Kα radiation, Ni filter. The powder samples were dropped onto silicon wafer with grease, and measured at the same equipment (5-80°, at a step of 0.0197°, with accumulation time 200 s per step). The morphology and porous nature characterized through scanning electron microscopy (FESEM-FEI Nova-Nano SEM-600) and transmission electron microscope (JEOL JEM-3010 with accelerating voltage at 300 kV). The Raman spectra were recorded in backscattering arrangement, using 532 nm laser excitation using 6 mW laser power. X-ray photoelectron spectroscopy (XPS) was performed on a PHI 5000 Versa Probe II scanning XPS microprobe from Physical Electronics, using X-ray radiation from an Al source equipped with a monochromator. Spectra were collected and evaluated with the MultiPak (ULVAC-PHI, Inc.) software. All binding energies were referenced to the C1s peak at 284.8 eV. The Raman spectrum of respective sample were collected through instrument, DXR Raman (Thermo, USA); laser wavelength: 633 nm, laser power on sample: 2mW, exposition time: 5s, 32 spectra were averaged at each spot to obtain one data point. Transmission electron microscopy (TEM) including high-resolution TEM analysis was performed using a FEI Tecnai G2 microscope. Selected-area electron diffraction patterns (SAED) were analyzed and fast Fourier transform (FFT) patternswere processed by using the Gatan Digital Micrograph software. Samples for AFM measurements were prepared by depositing a suspension in water on a mica surface. AFM images were taken ex-situ using a Nanowizard II (JPK Instruments) operating in tapping mode. All binding energies were referenced to the C1s peak at 284.8 eV. Adsorption studies of N_2 (77 K) of all samples were carried out using MICROMERITICS analyzer, outgassed at 423 K under high vacuum.

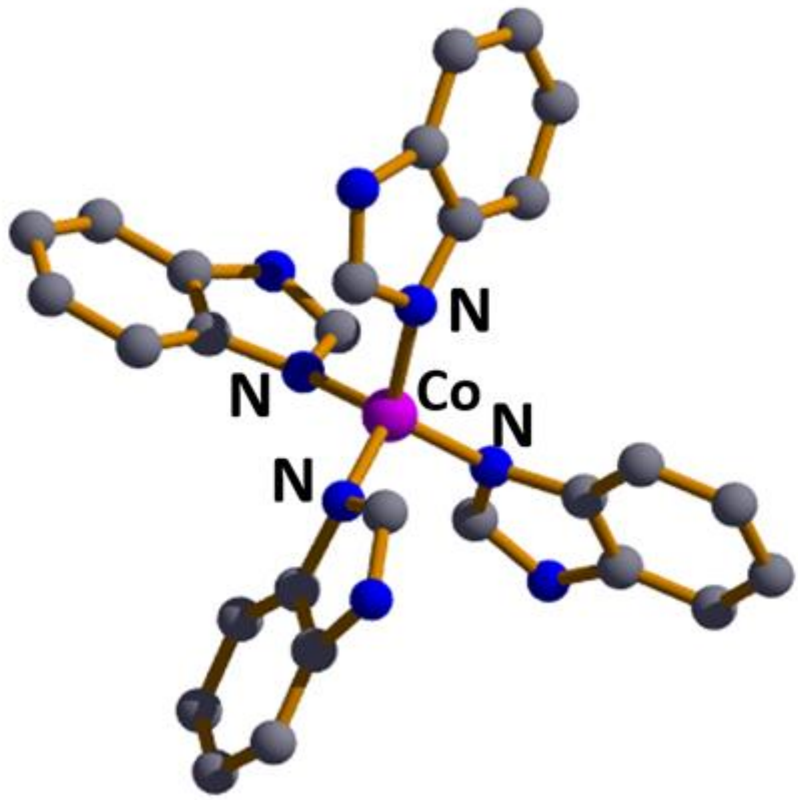

Figure S1. View of building unit of Co-ZIF-9-III phase, cobalt coordinated with benzimdazole groups presented with atom numbering.

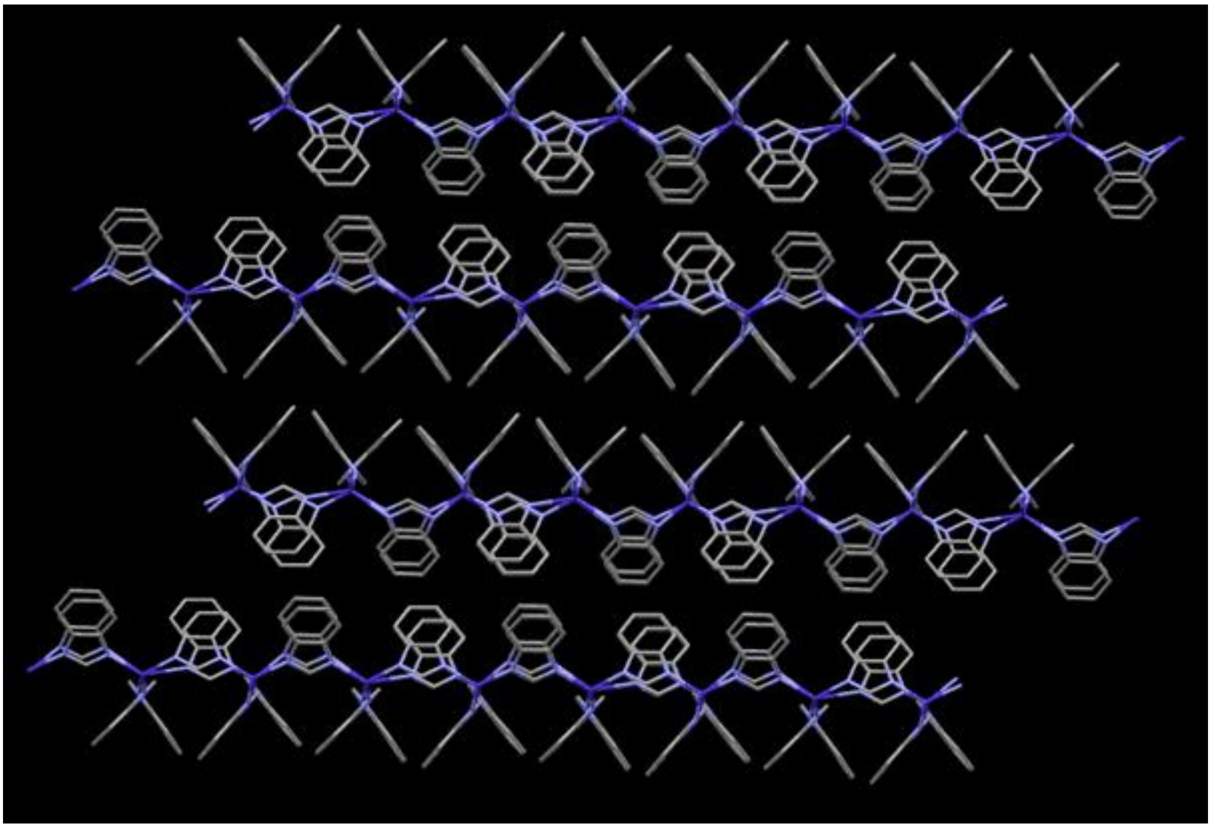

Figure S2. Three dimensional network of Co-ZIF-9-III lying in the crystallographic ab-plane. Further it gives information of individual layers with weak interactions (hydrogen, Vander walls forces)

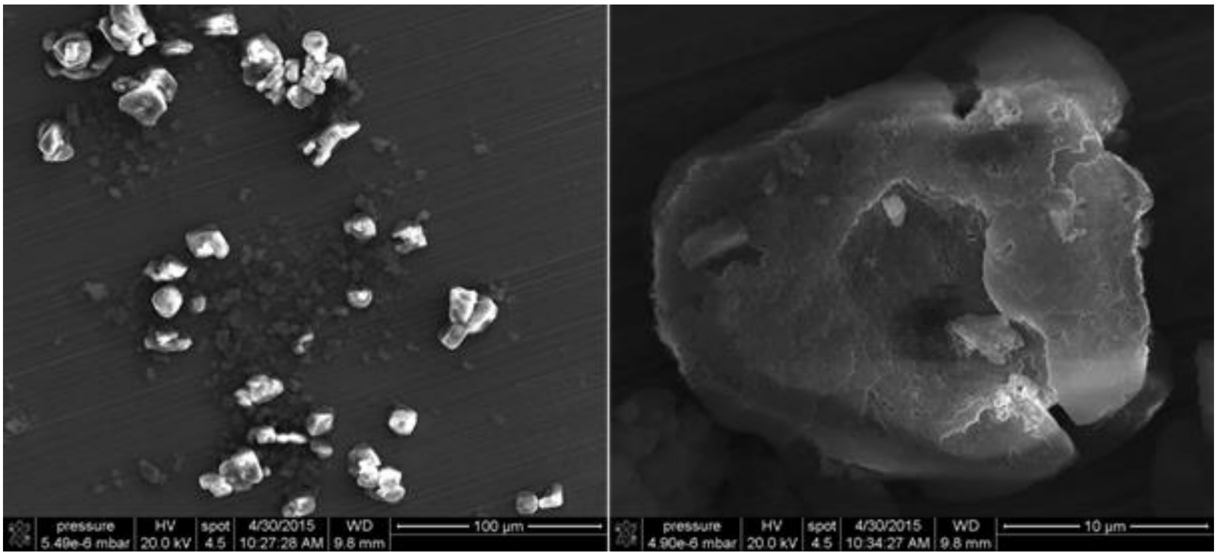

Figure S3. Low magnification of FESEM Image of mechanochemical synthesis of CoZIF-9-III materials, showing bundle of micro spheres and each sphere have regularly arranged nanoscale plates of CoZIF-9-III.

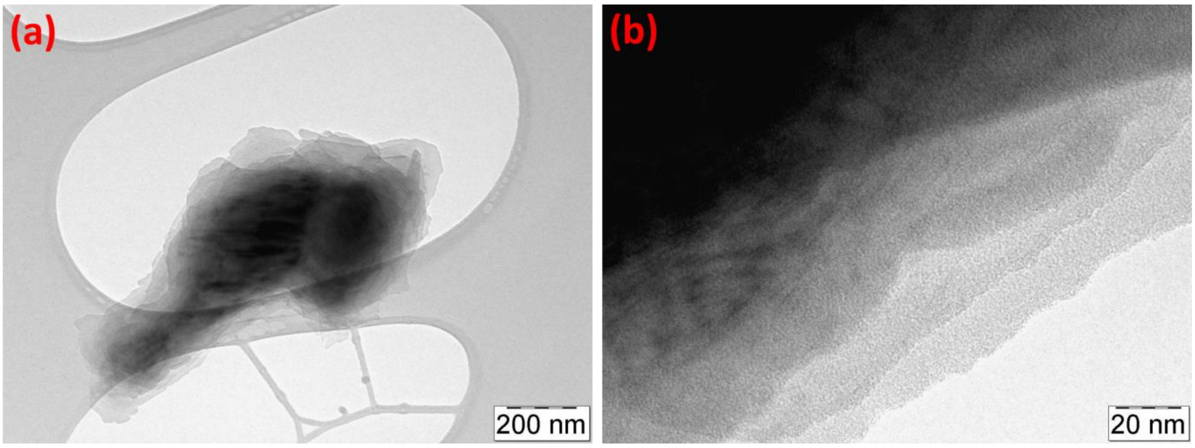

Figure S4. (a) TEM image of bulk Co-ZIF-9- III, and (b) a magnification of the image showing 3-4 nanosheets of nanometer thickness.

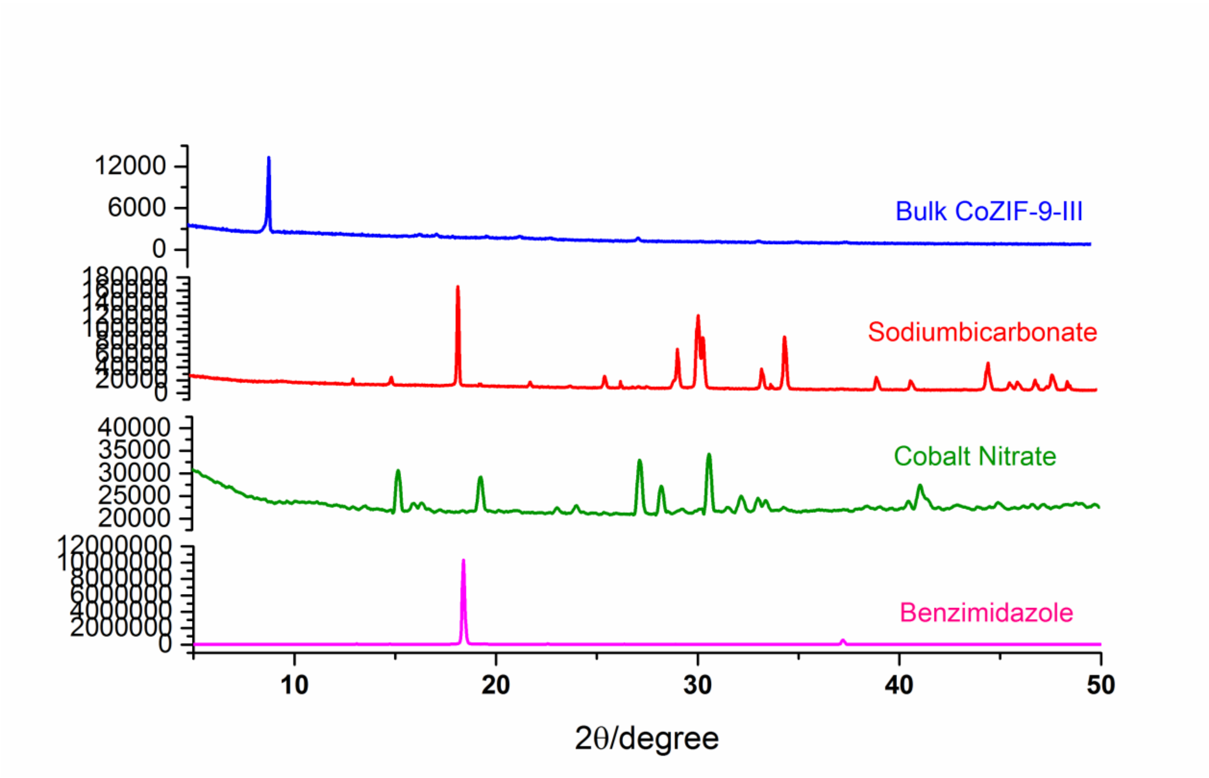

Figure S5a. Powder XRD patterns of the initial precursors, benzimidazole, cobalt nitrate, sodium bicarbonate under mechano-chemical synthesis of CoZIF-9-III. The comparative powder XRD patterns confirms there is no impurity of initial precursors present in the final product

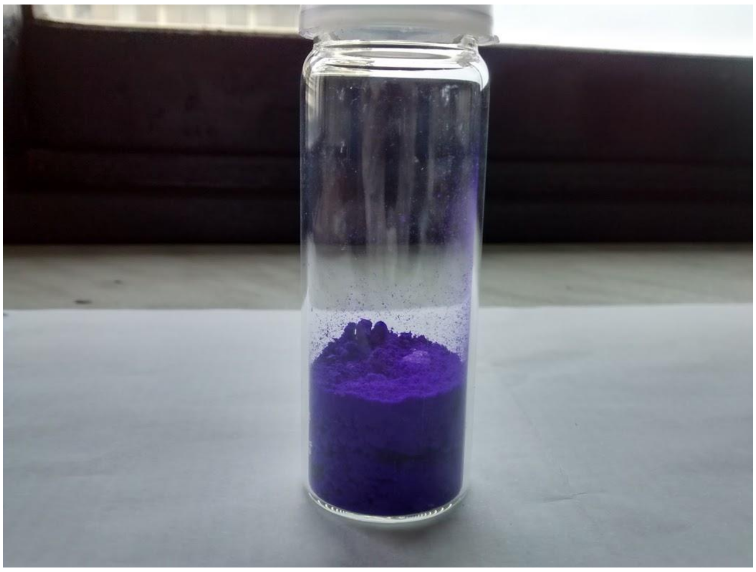

Figure S5b. Optical image of synthesized bulk Co-ZIF-9 material upon mechanochemical grinding method.

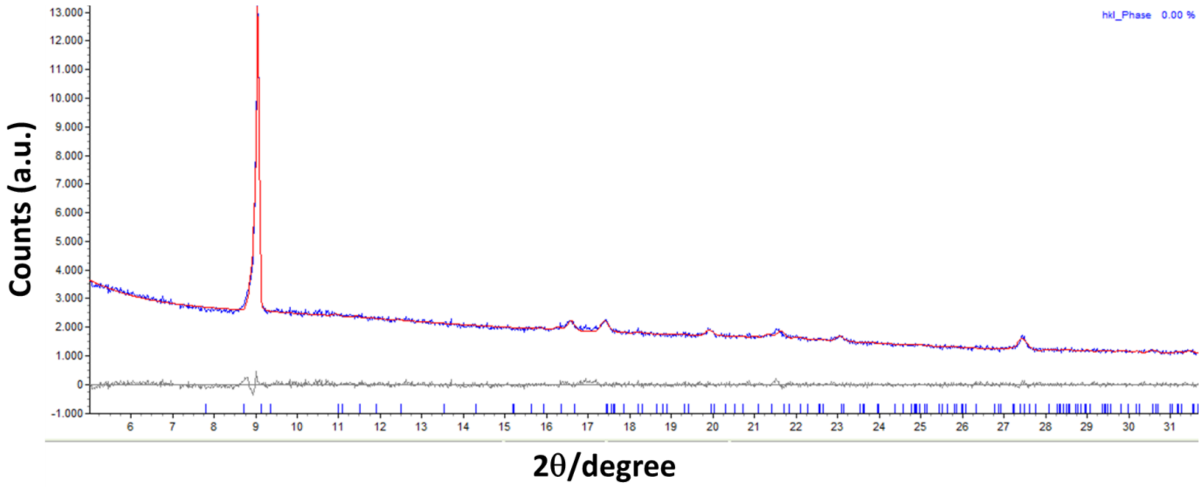

Figure S6. Powder XRD pattern for bulk Co-ZIF-9-III and Pawley fit. Blue-experimental, red-calculated, and grey-difference between experimental and calculated curves. The positions were intensities are expected are shown in blue.

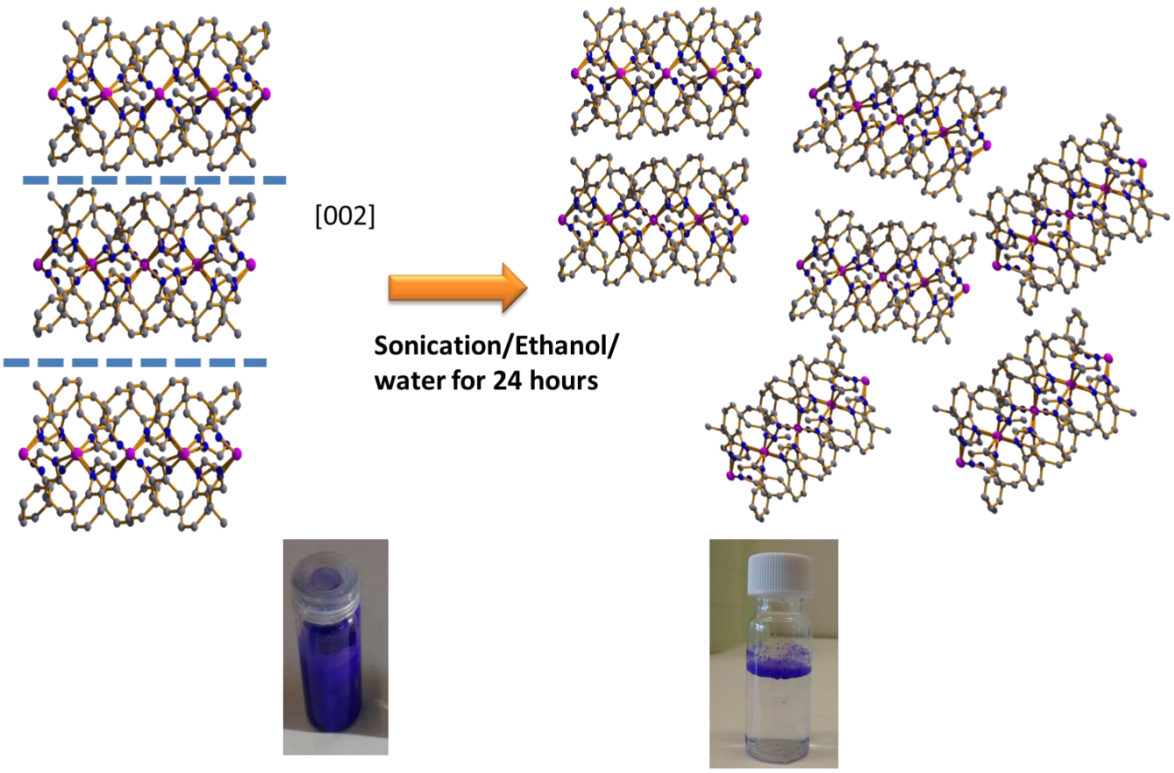

Figure S7. Schematic representation showing liquid-phase exfoliation (1:1 water/ethanol mixture) of bulk Co-ZIF-9(III) and corresponding optical images (left) of bulk Co-ZIF-9(III) in a water/ethanol mixture; and (right) some aggregated exfoliated nanosheet material floating on the diluted colloidal solution in water (see Figure S8). The scheme also reveals carination of some parts of the exfoliated layers in the (002) plane of the nanosheets.

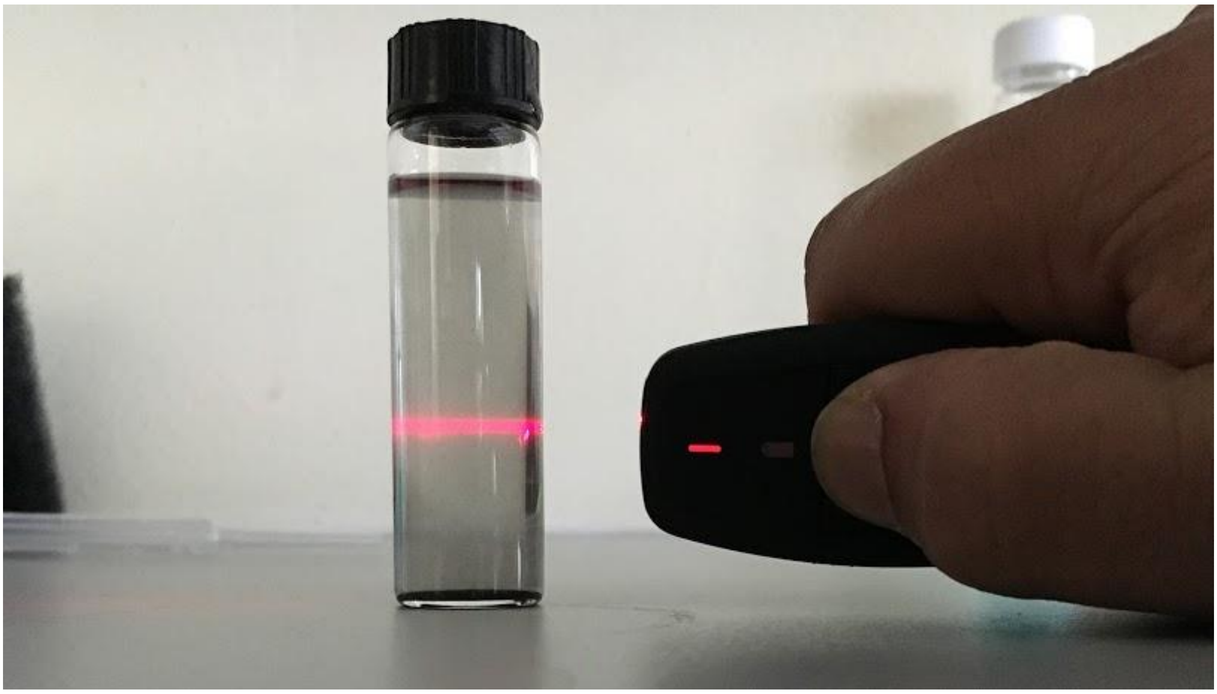

Figure S8. Colloidal dispersion of exfoliated Co-ZIF-9(III) nanosheets showing the Tyndall effect.

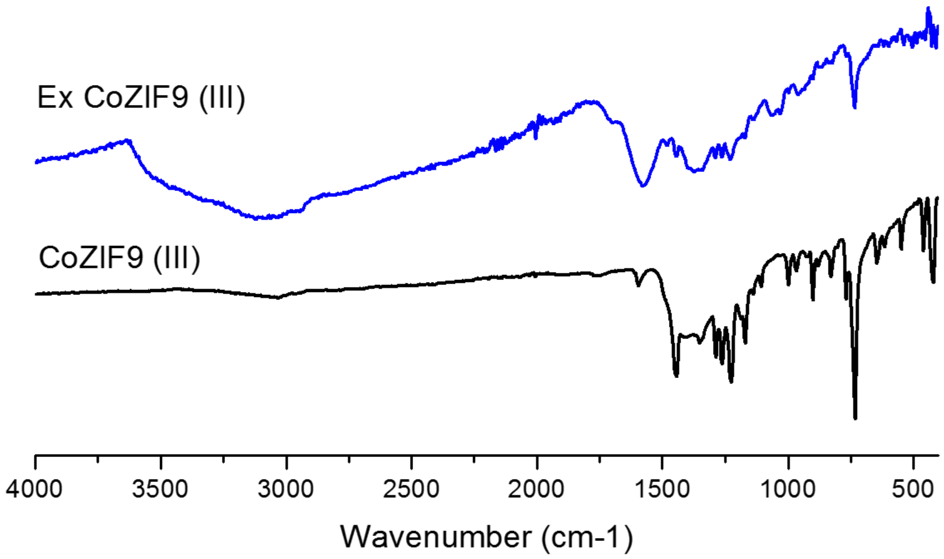

Figure S9. FT-IR spectra showing structural rigidity of Co-ZIF-9-III phase before and after liquid-phase exfoliation.

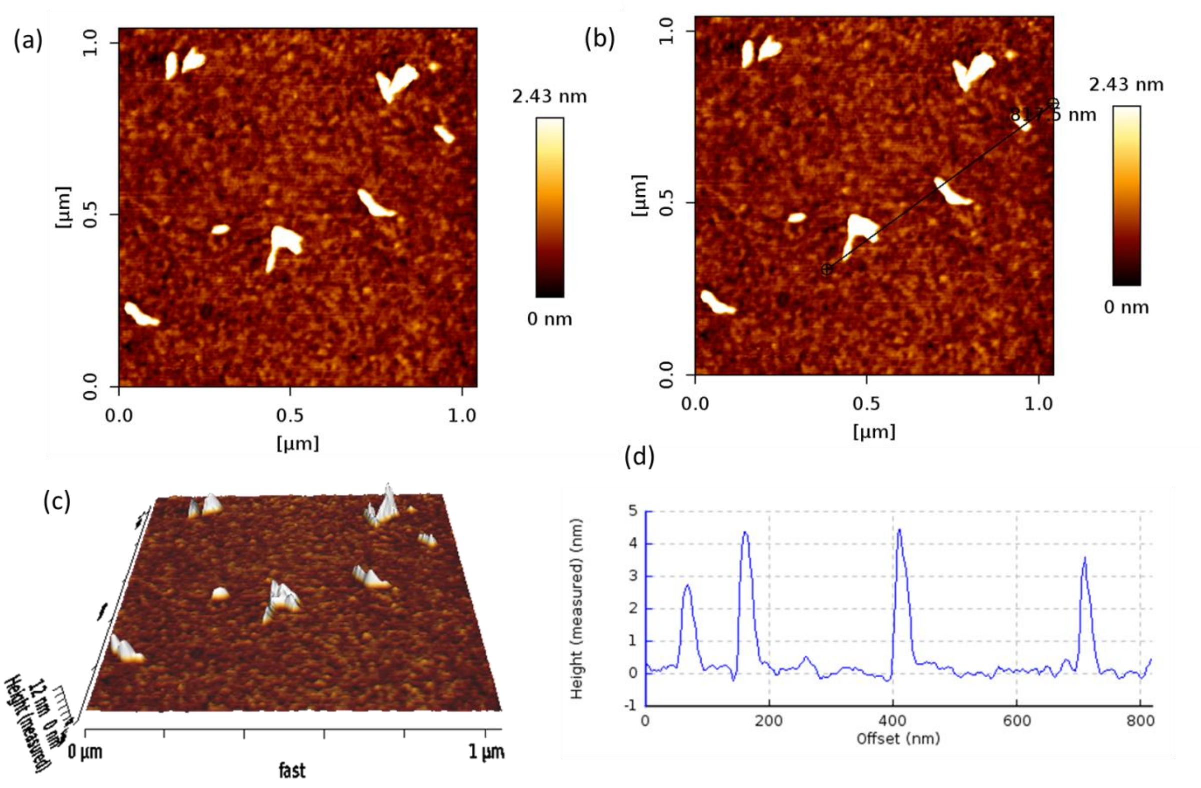

Figure S10. AFM topography showing several Co-ZIF-9(III) nanosheets with a thickness in the range of 2-4 nm.

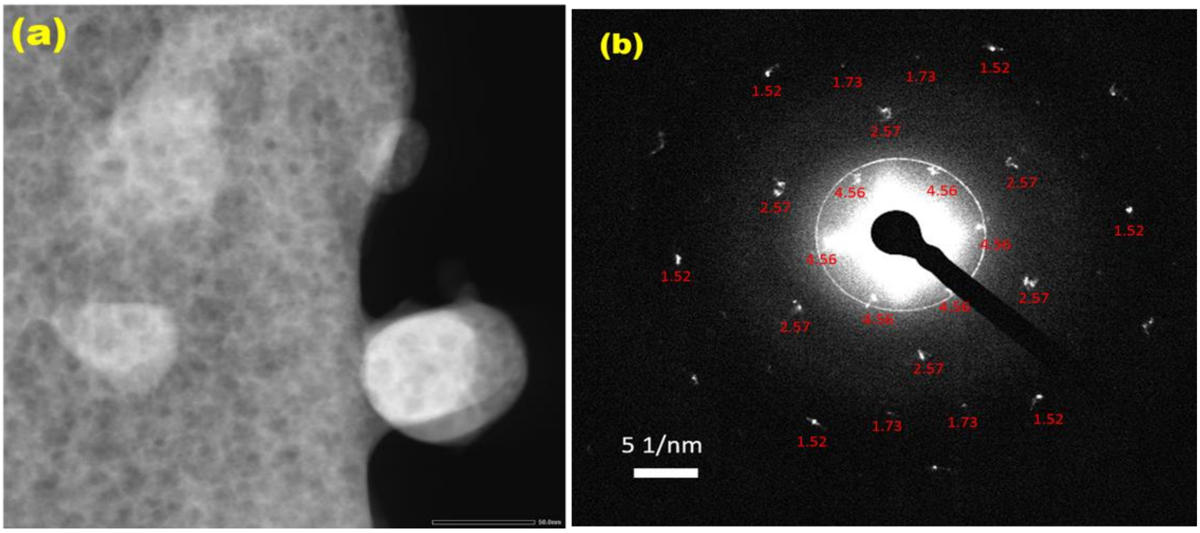

Figure S11. HRTEM image (a) and electron diffraction pattern (b) showing the crystalline nature of the CoZIF-9-III nanosheets.

The d spacings of 4.56 Å, 2.57 Å, and 1.52 Å are assigned to the $(13\underline{2})$, (532), $(39\underline{6})$ lattice planes, respectively, of the CoZIF-9-III nanosheets, thus revealing their crystalline structure. The diffraction spots showing irregular shapes instead of typical circular disks reflect the complicated structure itself along the projected direction. The white ring around the center is an artifact caused by electronic optics and should therefore be ignored. Due to the thin nature of the sample, the integration time of camera exposure had to be elongated, which causes the large bright area at the center of the diffraction image without affecting the observation of the spots.

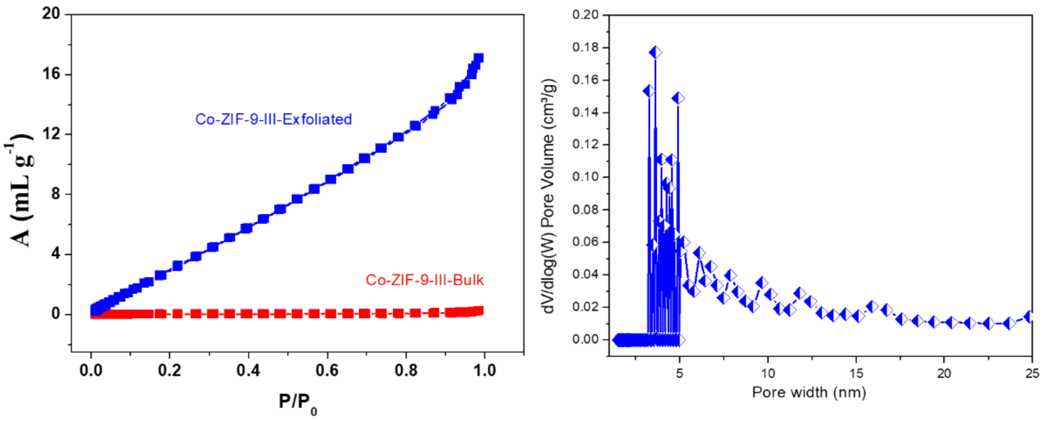

Figure S12a. (a) Nitrogen adsorption-desorption isotherms (left), and pore size distribution (right) calculated from the NLDFT method.

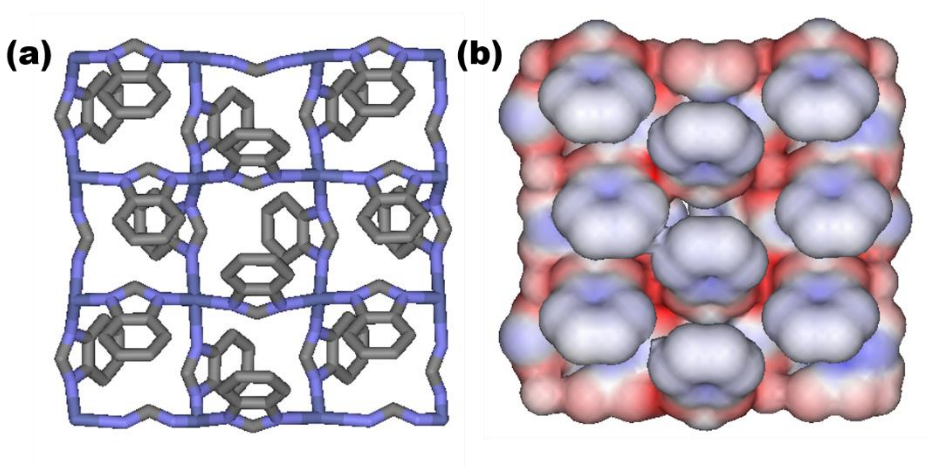

Figure S12b. View of two-dimensional network of CoZIF-9 (a) stick modal (b) surfaces provided to network.

The data of Figures S12 confirm the (expected) non porous nature of bulk CoZIF-9 powder and, however, the enhanced adsorption properties of exfoliated material with a specific surface area of 19 m^2/g. The pore size analysis confirms a mesoporous structure which is assigned to the disordered nano-/mesoscale aggregation of the exfoliated nanosheets.

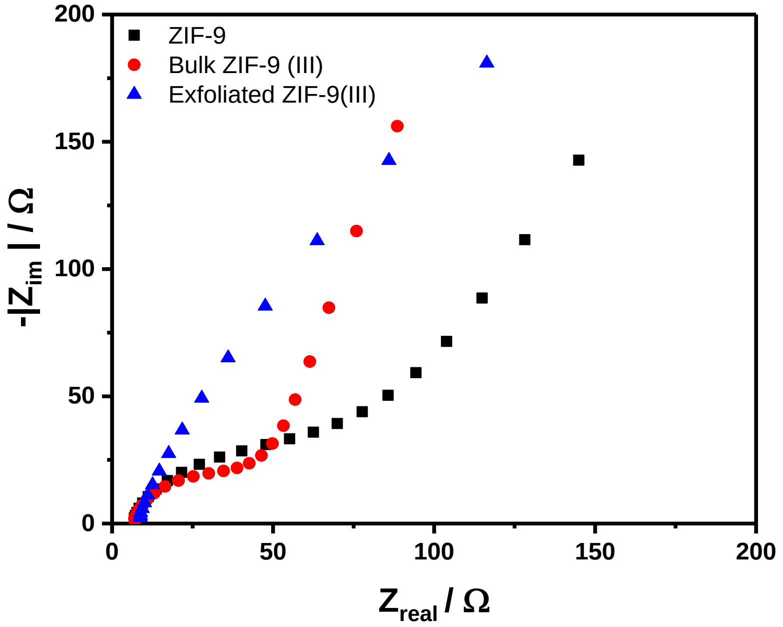

Figure S13. Nyquist plots showing the impedance of glassy carbon electrodes modified with Co-ZIF, bulk Co-ZIF9(III), and exfoliated Co-ZIF9(III) nanosheets. The spectra were recorded in 1.0 M KOH using ac perturbation of 10 mV (rms) in the frequency range from 50 kHz to 10 Hz at the respective open circuit potentials of the electrodes.

The charge transfer resistance across the electrode-electrolyte interface, determined from the diameter of the semi-circle from the Nyquist plots decreased in the order Co-ZIF9 (40.7 Ω > bulk ZIF9(III) (23 Ω) > exfoliated ZIF9(III) nanosheets ≈ 0 Ω. Therefore, besides the expected higher density of exposed active sites in the exfoliated 2D ZIF9(III) nanosheets compared to Co-ZIF9 and the bulk ZIF9(III), the negligible charge transfer resistance of the 2D ZIF9(III) nanosheets is suggested to also contribute to their superior electrocatalytic OER activity.

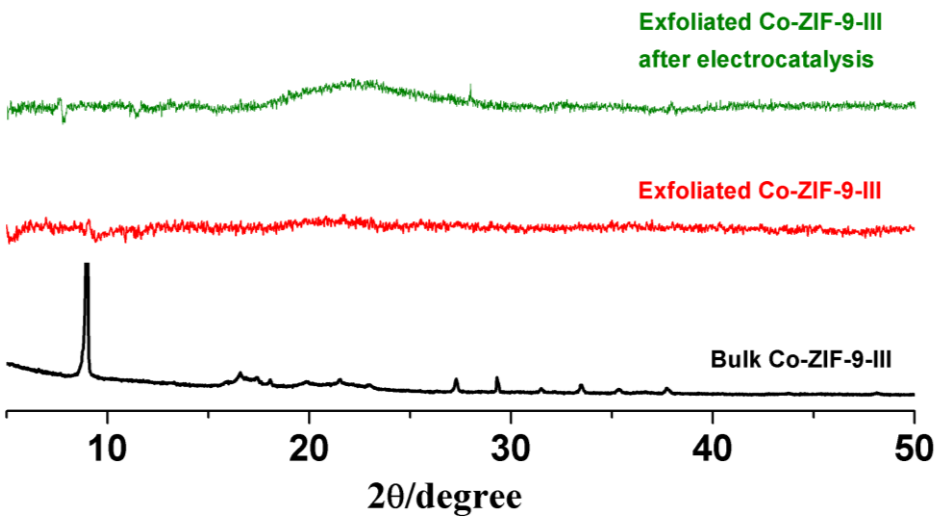

Figure S14. Powder XRD patterns of CoZIF-9-III phase bulk (black), exfoliated (red) and after the material after elctrocatalytic measurments (green). The broad peak (green trace) is corresponding to grease during powder XRD measurments (sample preparation).

The bulk Co-ZIF-9-III shows all peaks corresponding to ZIF-9-III phase (black line), which can be ascribed to the monoclinic structure of ZIF-9-III. After liquid exfoliated Co-ZIF-9-III (red line), some of the nanosheets lie on the solid substrate with (002) as the preferred orientation. A very weak 002 peak (red trace) is matching with the dominant 002 peak (black trace) of the bulk material. After electrocatalysis of oxygen evolution, we did not observe any peaks corresponding to the bulk materials, which may be due to randomly oriented Co-ZIF-9-III nanosheets. Moreover, after electrocatalysis we did no observe any XRD peaks from cobalt oxide phases. Note, that the amount of material deposited as coating to modify the electrode for electrocatalytic measurements is extreme lo. It is therefore not surprising that structural information is difficult to obtain by standard XRD techniques. The microstructural characterization of the local environment of the Co sites of the electrocatalyst materials is thus based on spectroscopic evidences (XPS, RAMAN etc.).

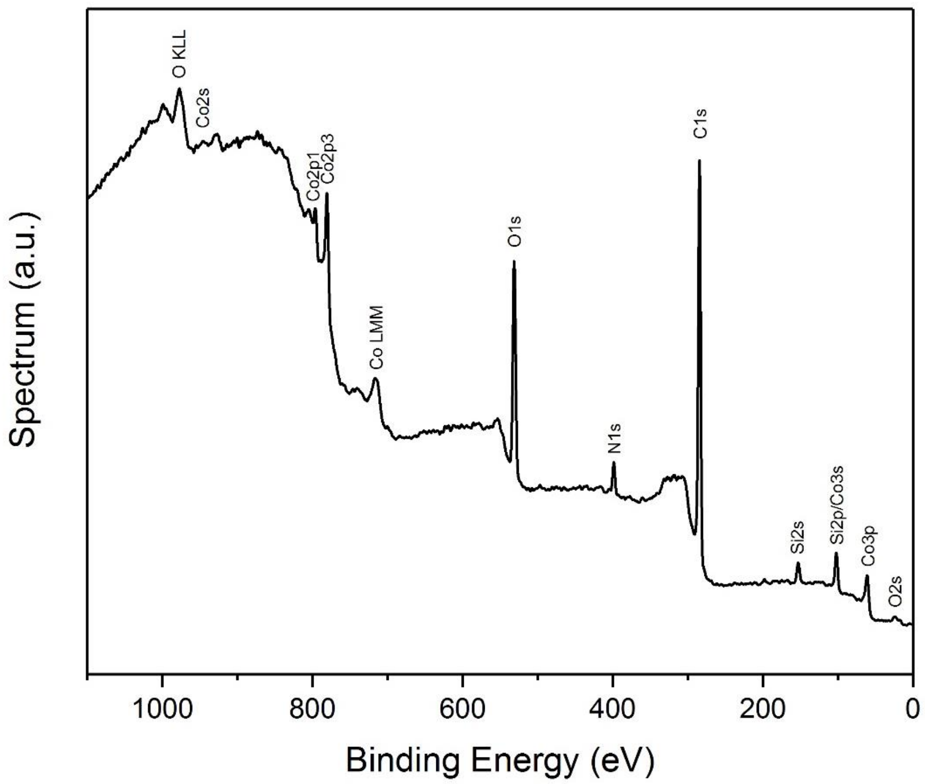

Figure S15. XPS analysis, survey spectrum of Co-ZIF-9-III sheets after electrochemical activation.

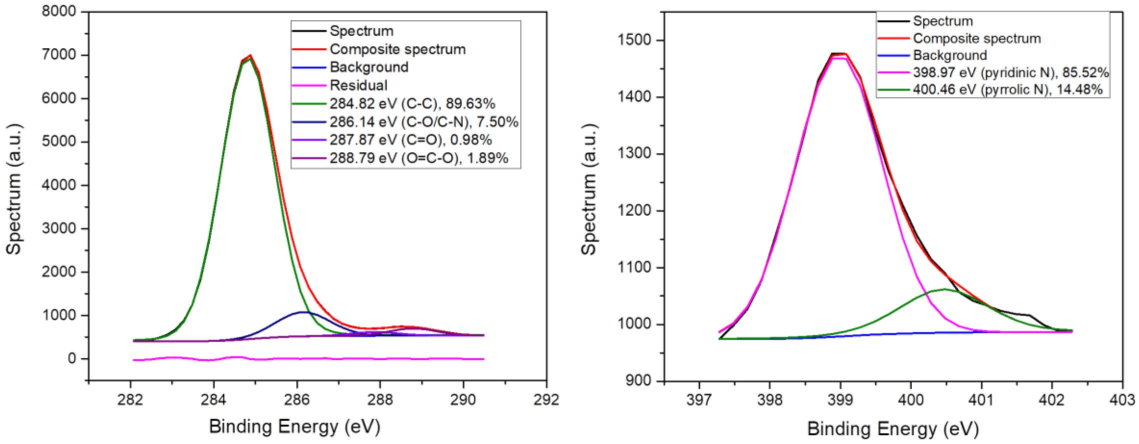

Figure S16. High resolution C 1s (left) and N 1s (right) XPS spectra of Co-ZIF-9-III sheets after electrochemical activation and continuous polarization at 10 mA cm^{-2} for 3 h.

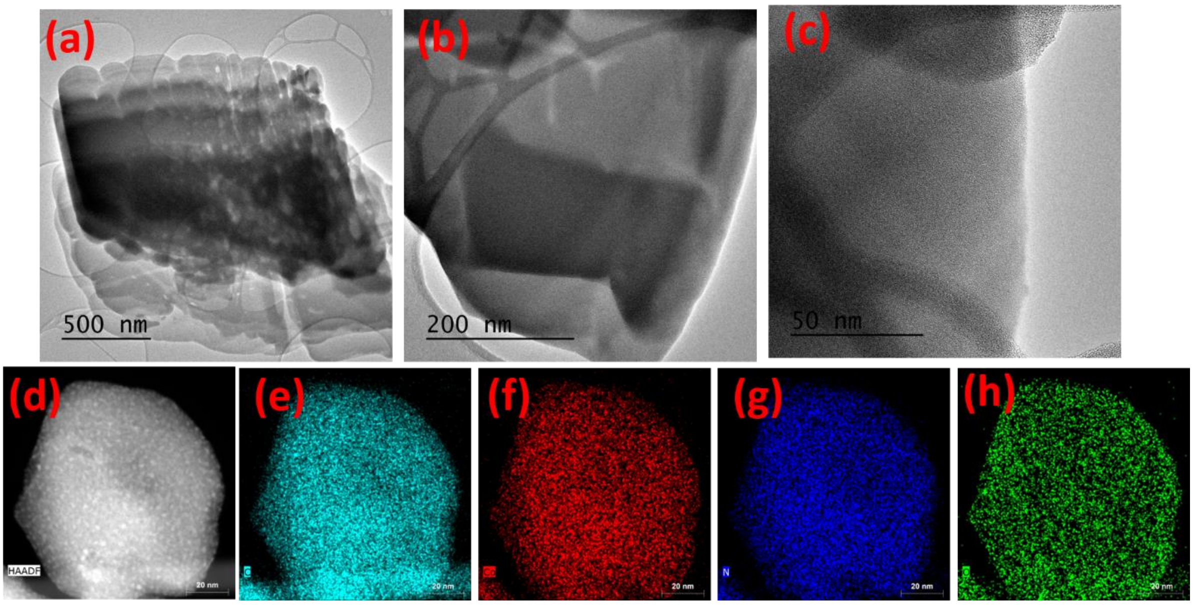

Figure S17. (a-c) HR TEM images of exfoliated Co-ZIF-9-III after catalysis showing its stable nanosheet morphology even after electrocatalytic measurements. (d-h) Elemental mapping showing homogeneous distribution of carbon, cobalt, nitrogen and oxygen elements respectively.
